# Experimental investigation the effect of bamboo micro filler on performance of bamboo-sisal-E-glass fiber-reinforced epoxy matrix hybrid composites

**DOI:** 10.1016/j.heliyon.2024.e40176

**Published:** 2024-11-06

**Authors:** Debela N. Gurmu, Hailemariam M. Gebrelibanos, Chala A. Tefera, Belete Sirahbizu

**Affiliations:** a Department of Materials Technologies, Faculty of Materials Engineering, Silesian University of Technology, Katowice, Poland; b Department of Mechanical Engineering, School of Mechanical and Industrial Engineering, Ambo University, Ambo, Ethiopia; c Department of Mechanical Engineering, Addis Ababa Science and Technology University, Addis Ababa, Ethiopia; d Xinjiang Technical Institute of Physics & Chemistry, Chinese Academy of Sciences, Xinjiang, China

**Keywords:** Bamboo micro filler, Sisal, Bamboo, Hybrid composite, Water absorption

## Abstract

The numerous advantages of natural fiber reinforced hybrid composites, such as their light weight, biodegradability, recyclability, availability, and low cost, have brought them to the forefront for various structural applications in the automotive and aerospace industries. Aim of this study was to evaluate the impact of varying the weight fractions of bamboo micro filler on the tensile, flexural, impact, and water absorption properties of the Sisal-Bamboo-E-glass fiber reinforced epoxy matrix hybrid composite prepared by manual hand layup method. To enhance the bond between natural and synthetic fibers and reduce the hydrophilic nature of natural fibers, bamboo and sisal fibers were treated with 5 % NaOH for 6 and 8 h, respectively. The E-glass fiber content was kept constant at 10 %, while the weight fractions of sisal, bamboo fiber, epoxy matrix, and bamboo micro filler were varied. The bamboo micro filler size ranged from 75 to 100 μm. Tensile and flexural tests were conducted using a computer-controlled universal electromechanical testing machine, while impact testing was performed with a Charpy impact test machine. The results showed that the hybrid composite containing 15 % sisal, 10 % glass, 15 % bamboo fiber, 57 % epoxy, and 3 % bamboo micro filler exhibited the highest tensile strength (87 MPa),flexural strength (77 MPa), high impact energy (8.9 J) and high toughness (24.64 J/cm^2^). Conversely, the highest water absorption capacity was observed in composites with 6 % bamboo micro filler. Overall, the tensile, flexural, impact, and water absorption properties of the hybrid composites were significantly influenced by the weight fractions of the fibers and bamboo micro filler.

## 1 Introduction

In recent times, composite materials have demonstrated their value across various industries, including sports, automotive, aerospace, and naval, due to their exceptional mechanical properties and lightweight nature [1]. The use of natural fibers as reinforcement materials has gained traction, particularly in the structural sectors of the global composite industry. This trend is driven by pollution concerns, the need for renewable resources, increased environmental awareness, and economic challenges. As a result, many engineering composite applications are now utilizing natural fiber resources instead of synthetic ones [2]. Natural fibers, which come from bio-based sources such as plants and animals or are found as minerals in nature, offer an alternative to synthetic fibers [3]. These natural fibers can be categorized into three main types: animal-based (e.g., silk, wool, and hair), mineral-derived (e.g., asbestos, wollastonite), and plant-based lignocellulosic fibers (e.g., hemp, flax, jute, sisal, palm fiber, kenaf, date palm, etc.) [4,5]. Natural fibers are known for their biodegradability, recyclability, renewability, and environmental friendliness. However, compared to synthetic fibers like glass and carbon fibers, natural fibers are less durable, have weaker interfacial bonding, lower strength, and higher water absorption [6,7].

Consequently, many researchers are exploring innovative methods to enhance the properties and performance of natural fiber composites. One approach that can improve the mechanical, thermal, and dynamic properties of composites is hybridization [8]. This technique involves combining natural and synthetic fibers to create composite materials with desirable mechanical performance [9]. The primary goal of hybridization is to merge two or more components to produce a product that exhibits superior features or performance for its intended use compared to the individual materials. The process of blending two or more fibers into a polymeric material to address the shortcomings in mechanical and other attributes of other fiber materials is known as the “hybridization of composite materials” [10]. This combination of natural and synthetic fibers in hybrids is an effective way to reduce material costs and mitigate environmental impact. Additionally, hybridization addresses issues such as the non-biodegradability and high cost of synthetic fiber reinforcement, as well as the lower mechanical properties and high-water absorption capacity of natural fibers. The hybridization of synthetic and natural fibers has been extensively studied. For example, Gurmu et al. [11] investigated the mechanical properties of E-glass/sisal fiber reinforced epoxy matrix composites, including tensile, compressive, and flexural characteristics. The authors found that incorporating synthetic fiber into the composites significantly enhanced the mechanical properties of natural fibers. Additionally, Chethan et al. [12] developed a hybrid composite consisting of kenaf, jute, and E-glass with epoxy resin as the matrix and studied its mechanical properties. The authors suggest that hybrid laminates incorporating kenaf and glass fibers can enhance the mechanical properties of composites compared to those made solely with jute and glass fibers. Conversely, Sanjay et al. [13] and Gadisa et al. [14]studied the tensile, flexural, impact, and interlaminar shear strengths of Jute-E glass hybrid composites. Results indicate that the hybrid composite exhibits significantly superior characteristics compared to pure jute fibers.

Purohit et al. [15] investigated how fiber orientation and weight fraction influence mechanical properties (tensile and flexural strength), erosion wear characteristics, and various physical criteria (density, void content, and water absorption behavior). The experimental results indicate that the rate of water absorption increases progressively with higher fiber loading. Additionally, according to a study by El-baky [16], the amount of fiber content, the stacking order of the fibers, and the effect of hybridization on the tensile and flexural properties of glass, jute, and carbon fiber were examined. The authors found that adding glass and/or carbon fiber enhances the low tensile and flexural properties of pure jute fiber composites. When glass and/or carbon fibers are hybridized with jute-reinforced composites, the void content decreases. Compared to other composites containing glass and/or carbon fibers, pure jute/epoxy composites exhibit the highest void content because glass and/or carbon fiber mats are more compatible with epoxy resin than jute mats. Optimal flexural strength and modulus are achieved when carbon fiber layers are stacked at the exterior edges of the composite. Conversely, composites (JGCs) with jute fiber layers stacked on the exterior show the lowest flexural strength and modulus. Furthermore, Thandavamoorthy et al. [17]) studied the mechanical and water-absorbing properties of hybrid composites. They found that the tensile, flexural, and impact properties of fiber hybrid polymer composites are influenced by the stacking order.

In the current context, due to their biochemical composition, natural reinforced fibers are hydrophilic and require treatment to improve compatibility with hydrophobic thermoplastic and thermoset matrices. Treatments include chemical (alkaline, silane, acetylation, etc.), biological, physical (enzyme), thermal, benzoylation, permanganate, and peroxide treatments [[18], [19], [20], [21]]. Among these, alkaline treatment with NaOH is the most favored due to its effectiveness and low cost [22]. This treatment is used to reduce the water absorption rate through surface modification [23]. Numerous articles discuss various natural fiber treatments and their impact on the performance of composite materials. For instance, Neto et al. [24] studied the effect of chemical treatment on the thermal properties of hybrid natural fiber (jute, ramie, and sisal) reinforced polymer matrix composites (NFRC). The authors found that chemical treatments enhanced thermal stability and altered the morphology of natural fibers. Raju et al.[25] investigated the effect of alkali treatment on the physical-chemical, tensile, thermal, and surface properties of Symphirema involucratum stem fiber (SISF). The treated fiber was deemed suitable for lightweight applications, as physical analysis showed an increase in fiber density to 1424 kg/m³ after surface treatment, reducing the overall weight percentage. Enhancements in tensile strength (471.2 ± 19.8 MPa), tensile modulus (5.82 ± 0.77 GPa), and thermal stability (371 °C) were observed, indicating that the treated fiber possesses the necessary mechanical and thermal properties for composite preparation.

The objective of this paper was to determine the effect of bamboo micro fillers on the mechanical properties of bamboo-sisal-E-glass fiber-reinforced epoxy matrix hybrid composites. Uniaxial tensile testing, flexural testing, impact resistance testing and water absorption properties measurement of hybrid composites contained a different weight fraction of bamboo micro-filler (0,3 and 6 %) were conducted experimentally. Through the study of these properties, the research contributes to the development of lightweight, eco-friendly composites suitable for applications in industries such as automotive and construction. This combination of natural and synthetic fibers, along with microscale fillers, provides a novel approach that has not been extensively covered in existing literature.

## 2 Materials and methods

### 2.1 Materials

Bamboo, sisal, and E-glass fibers were used as reinforcement in this article. Bamboo micro filler and epoxy resin were purchased from World Fiber Glass and Waterproofing Engineering, Addis Ababa, Ethiopia. Bamboo has got a lot of attention as a potential ecological structural material because of its wide distribution (more than 1000 species), short growth cycle (3–5 years), high stiffness, and superior fiber strength [26]. In contrast, sisal fiber was obtained from Addis Ababa Science and Technology University and removed manually. Sisal fiber has a lot of promise for reinforcing composite materials because it is inexpensive, low density, has a high modulus and specific strength, has minimal health risks, is readily available in some areas, and is renewable [27].

**Fig. 1:**
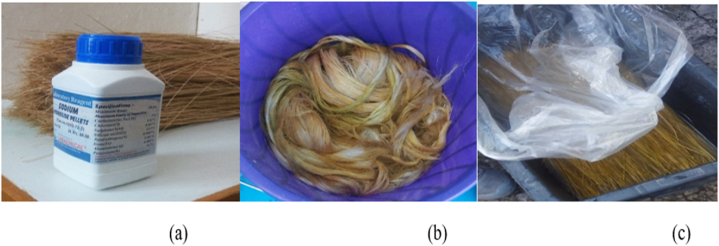
(a) Sodium hydro-oxide, (b) sisal fiber, and (c) Bamboo fiber.

**Fig. 2:**
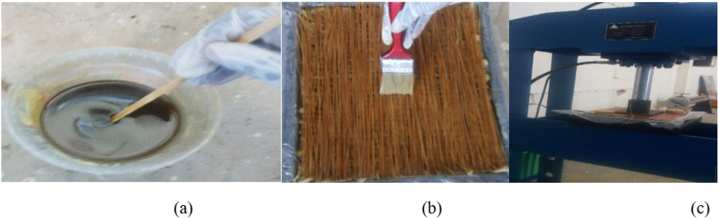
(a) Mixing filler with epoxy matrix, (b) spraying epoxy on hybrid composite, and (c) curing under a hydraulic press machine.

The detail sisal fiber extraction and sisal fiber is available in Fig. 1, Fig. 2 of paper [11]. Furthermore, properties like,high strength, corrosion resistance, and light weight make E-glass most suitable from other composite fiber [19]. Epoxy resin AY-105, Brand name “SYSTEM # 2000 EPOXY,” was acquired from Kadisco Paint and the adhesive industry, Addis Ababa, Ethiopia, and utilized as the matrix material in this research. The hybrid composites were cured using KEY CCB as the hardener.

. The purpose of adding bamboo micro filler to the composites is to increase the matrix's elastic modulus and impact strength, replace synthetic fillers, reduce shrinkage after curing, increase the matrix's fracture toughness, which in turn improves the mechanical properties of hybrid composites. Additionally, bamboo filler particles are utilized in the creation of hybrid composites because of their promising qualities, which include ease of handling, no orientation issues, minimal defects, and affordability [28]. Mold releasing agent (brand name HONEY WAX 250), which is utilized in this study was purchased locally from World Glass Fiber and Water Proofing PLC, may avoid samples from adhering during sample fabrication. Finally, for natural fiber treatments, NaOH and distilled water obtained from the atomic laboratory and the PLC teaching materials, Kirkos sub city, Addis Ababa were employed.

### 2.2 Sample preparation methods

The bamboo used in this paper was cut out and then splint into strips. The strips were then immersed in water for 12 h to eliminate any impurities that could have stuck during the extraction procedure [29]. The fibers were then manually removed, with an average thickness of 0.457 mm, as seen in Fig. 1a. Due to specific limitations of natural fibers when paired with synthetic fibers (carbon, aramid, and E-glass), such as a high degree of moisture absorption and a lack of affinity between the fiber and the matrix, natural fibers were treated. Hemicellulose and lignin concentrations in fibers are examples of polar components that are another reason why these materials must be treated before being used for a particular purpose [30]. Furthermore, there are several benefits to treating natural fibers, such as altering their crystalline structure, decreasing their hydrophilic qualities, strengthening the interfacial bond between the fibers and the matrix, decreasing the fibers' capacity to absorb moisture, and eliminating weak elements like lignin and hemicelluloses from their structure (31, 23). NaOH is the most widely utilized chemical treatment method for natural fibers due to its inexpensive cost, great impact strength, increased mechanical properties, good modification features, and wide availability. The proportion of NaOH combined with distilled water must be carefully considered when using natural fibers. This is because the mechanical characteristics of natural fibers are influenced by the proportion of alkali content. A 5 % concentration of sodium hydroxide was used, which seemed to guarantee improved raw fiber qualities. This is because different natural plant fibers can have their surface treated with 5 % sodium hydroxide to improve their mechanical, thermal, crystalline, and surface qualities (25). By decreasing the adhesive and bonding ability during the creation of composite samples, an increase in the percentage of NaOH has an impact on the fibers' characteristics[31]After 4 h of stirring in a solution of 2 % NaOH and 98 % distilled water, bamboo sawdust was ground into microparticles with a mesh size of 75–100 μm.

### 2.3 Sample preparation procedure

After collecting all materials required to make hybrid composites, bamboo and sisal fibers were treated with a 5 % alkaline solution for 8 and 6 h, respectively, as shown in Fig. 2 to modify the surface and ensure better fiber-matrix adhesion [[32], [33], [34]]. A mold made of 300 × 300mm flat steel and four RHS steel with a smooth flat wood cover was prepared to manufacture the hybrid composites. The sisal and bamboo fibers were oriented in 0° (unidirectional orientation),the E glass has a woven arrangement and bamboo micro-filler in prticulate orientation (Table 1). The percentage of weight fractions of selected fibers was shown in a mold that was cleaned and painted with release paste wax for the purpose of easy removal of fabricated hybrid composites. The matrix was applied to the surface with the help of a brush and a step-by-step stacking was performed employing a roller brush to remove excess matrix and air bubbles. Finally, the composition was compressed with 5 MPa pressure on a hydraulic press machine for 24 h as shown in Fig. 2c to cure the hybrid composites. The sample was prepared at room temperature around 23^0^ C.

**Table 1:** Experimental design.

Hybrid	Reinforcements (wt.%)			Matrix (wt.%)	
	Sisal	Bambo	E-glass	Epoxy	Micro-Filler
H1	10	20	10	60	0
H2	15	15	10	57	3
H3	20	10	10	54	6
Fiber orientation	Unidirectional (0°)	Unidirectional (0°)	Woven (0° and 90°)	–	Particulate

### 2.4 Hybrid composites

#### 2.4.1 Tensile test

**Fig. 3:**
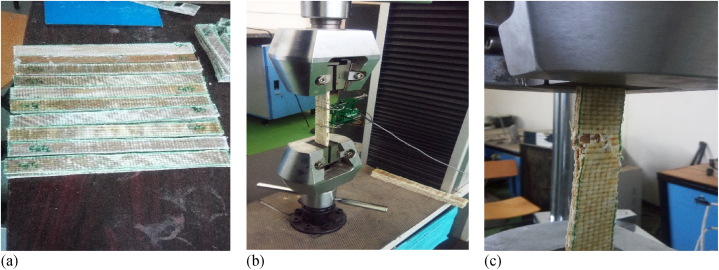
(a) Samples for the tensile test, (b) arrangement of the tensile test, (c) fractured sample.

The hybrid composite material manufactured from bamboo micro filler, sisal, E-glass and bamboo fiber were prepared for tensile test according to ASTM D3039 standard (Table 2). Three samples were prepared, and tensile test was performed on a computer controlled universal electromechanically machine as shown Fig. 3b. To increase the accuracy of tensile test, three samples were tested, and its average was used in this article.

**Table 2:** Summary of ASTM standard size of samples.

S/N	Tests	Dimension of Sample (width × length × thickness) in mm	ASTM Standards	References
1	Tensile	250 × 25 × 4	ASTMD3039	[11]
2	Flexural	120 × 20 × 3	ASTMD790-17	[36]
3	Impact	63 × 13 × 3	ASTMD256-10	[36]
4	Water Absorption	30 × 25 × 4	ASTMD570	[37]

#### 2.4.2 Flexural test

**Fig. 4:**
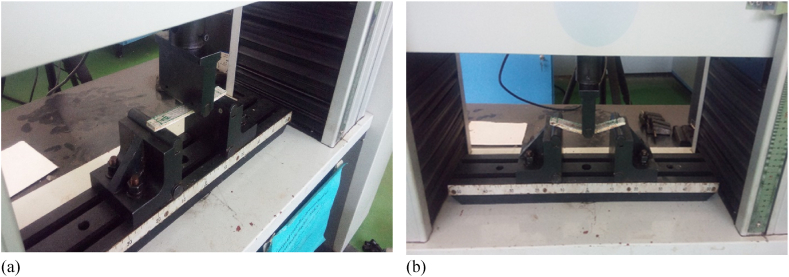
Flexural test arrangement, (a) pre-test and (b) post a test.

In this article flexural test (3-point bending) of hybrid composite have span length of 126 mm with offset cantilever length of 12 mm were prepared as per as ASTM D790-17 (Table 2). Flexural tests were also performed on computer controlled universal electromechanically Machine (Fig. 4). The test was done at test speed of 5 mm/min. The flexural strength of hybrid samples of three samples were calculated and its average value was used for further analysis.

#### 2.4.3 Impact test

**Fig. 5:**
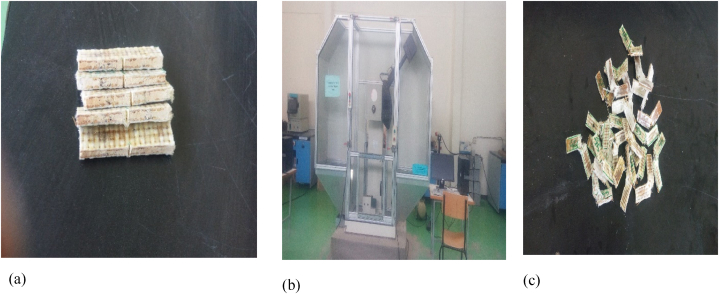
(a)Samples for the Charpy impact test, (b) Charpy impact test machine test, (c) sample after impact test.

For the Charpy impact test, samples were prepared in as per as ASTM D256-10 standard (Table 2) and notched to create a stress concentration. Using a computer-controlled Charpy impact testing machine, the impact energy absorbed by each sample was recorded for three types of hybrid composites with different weight percentages of bamboo and sisal fibers, along with varying weight fractions of bamboo micro filler (Fig. 5).

#### 2.4.4 Water absorption test

**Fig. 6:**
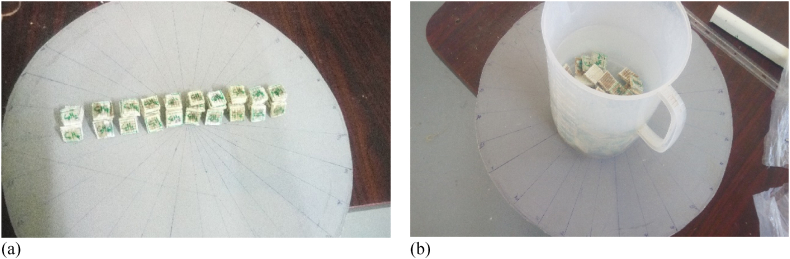
(a) Samples for water absorption, (b) Samples immersed in water.

The water absorptions were prepared according to ASTM D570 (Table 2) and immersed in water for one day to evaluate the hydrophilic properties of the hybrid composites. All dry and wet weights of five samples were recorded for each hybrid compound. Finally, the percentage of water absorption was calculated by equation 1 [35].

(1) $$\%\;\text{of}\;\text{water}\;\text{absorbtion}=\left(\frac{w_{f}-w_{i}}{w_{i}}\right)$$

where w_f_ is the weight of it before immersing the sample and w_i_ is the weight of the samples after removing it from the water (see Fig. 6).

## 3 Results and discussion

### 3.1 Tensile properties

**Fig. 7:**
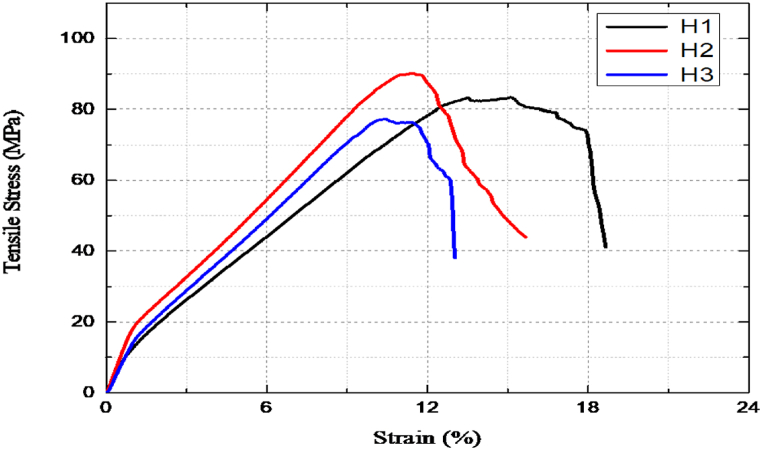
Stress-strain diagram for hybrid composites.

In longitudinal tensile tests using a universal test machine, the basic parameters recorded are stress and stress. However, the values of the parameter recorded for all samples were not the same because of various factors such as the testing environment, effect of temperature, manufacturing defects (the samples were manufactured by hand), and human errors. In addition to the weight fraction of the matrix (epoxy and bamboo micro filler), the weight fraction of sisal and bamboo fiber has a negative impact on the tensile strength of the E glass, sisal, and bamboo reinforced epoxy/bamboo micro filler matrix hybrid composite, as seen in Fig. 7. The reason for this might be that the tensile strength of composites reinforced with natural fibers varies depending on the fibers' size and type, the matrix material employed, and the production process [38]. When the weight fraction of the bamboo micro filler rose in these circumstances, the maximum tensile strength increased up to 3 % of the weight fraction of the bamboo micro filler before gradually beginning to drop. Result reported by Paul et al.[28] andGouda et al.[39] support this investigation, which states that the hybrid composite's ultimate tensile strength rises with an increase in the weight fraction of bamboo filler before beginning to fall with a larger weight fraction of bamboo micro filler. This results from the bamboo micro filler particles not being sufficiently moist and clumping. Additionally, because bamboo particles are poorly dispersed, adequate connection between the particulate and matrix is limited with increased filler loading, resulting in a stress concentration effect. As a result, stress is not efficiently transmitted from the matrix to the filler, which lowers the composites' tensile strength [40]. Furthermore, the maximum and minimum tensile strength were exceeded in hybrid composite 2 (15 % sisal and bamboo fiber each,10 % Eglass, 57 % epoxy and 3 % bamboo micro filler) and hybrid composite 3 (H_3_), respectively. A The maximum tensile strength of the hybrid compound with 3 % bamboo micro filler was 4.6 % higher than that of the hybrid compound 1 (0 %) bamboo micro filler). However, the maximum tensile load of the hybrid compound with 3 % bamboo micro filler was 6.1 % higher than of hybrid compound1 (0 %) of bamboo micro filler).

### 3.2 Flexural test

**Fig. 8:**
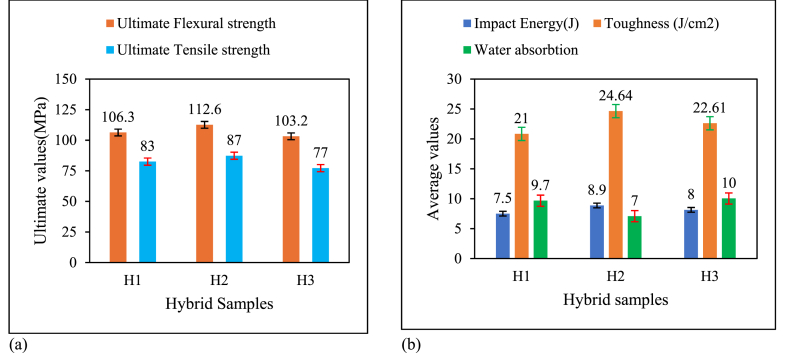
Flexural and tensile properties of hybrid composite(a), Impact energy, Toughness and water absorption values.

Flexural strength is defined as ability of a material to support bending force without breaking [38]. As shown in Fig. 8a, flexural strength was highly affected by weight fraction of the fibers, matrix and bamboo micro filler. Flexural strength increased first by 5.06 % as the weight fraction increased from 0 to 3 %. Then, flexural strength begins to decrease after weight fraction exceeds 3 %. Higher filler loading leads to poor polymer wetting, which causes degradation of flexural strength [28]. Furthermore, the expansion of cluster formation caused by the additional weight percentage of filler encircled the resin, resulting in a gap between the resin and hardener [41]. Due to the hydrophilic and hydrophobic characteristics of both the fiber and the matrix, the natural fiber's sticky ability may also be the cause of the strength reduction [42]. The strength of the hybrid composite is decreased by the rupture of the fiber-matrix bond brought on by the development of significant localized strain in the matrix [43]. Specifically, the increase of the weight fraction of bamboo micro filler from 3 to 6 % results in a decrease in flexural strength of 8.3 % and 25.6 % respectively. Furthermore, maximum flexural strength was observed in a hybrid composite 2(H_2_) with 15 % sisal, 15 % bamboo,10 % E-glass and 57 % epoxy and 3 % bamboo micro filler. Its values were 112.6 MPa from this result, the optimum weight fraction of bamboo micro filler was 3 %

### 3.3 Impact test

From the results of the Charpy impact test, impact energy of hybrid composite first increased as the weight fraction of bamboo micro filler increased from 0 to 3 %. After that, impact energy starts to decrease as the weight fraction of bamboo micro filler increased from 3 to 6 %. Initially, as the bamboo filler content increases from 0 to 3 %, there is likely an improvement in filler-epoxy matrix bonding, enhancing the hybrid composite's ability to absorb and dissipate impact energy. This improved adhesion facilitates better stress transfer, allowing the material to absorb more energy effectively. However, when the filler content surpasses 3 %, the impact energy begins to decline, potentially due to filler agglomeration or clustering, which can create weak points within the composite. These clusters reduce the material's uniformity, leading to lower energy absorption and impact resistance. Additionally, as the bamboo filler content increases further, the composite may exhibit increased brittleness. Higher filler concentrations can reduce the composite's ductility, making it more prone to cracking upon impact rather than deforming plastically, which diminishes its impact energy.

### 3.4 Water absorption

Fig. 8b illustrates how the water absorption characteristics of hybrid composites improved when the weight percentage of bamboo microfiber increased over 3 %. This is because water absorption capacity of a composite material increases with weight percentage of natural fiber[44] This may be due to lower bonding between fibers and increased void formation. Water absorption values were highest and lowest in hybrid composites containing 6 % and 3 % bamboo micro filler, respectively. Adding more bamboo micro filler above 3 % results in increase water absorption. This is because water absorption capacity is greatly affected by the natural fiber amount in composites [45] (see Table 2).

The tensile and flexural strengths are compiled in Table 3 and compared with previous published research to validate the findings of this investigation. When the weight fraction of bamboo micro filler increases (from 0 to 3 % in this work and 0–5 % in previous work), the tensile and flexural strengths of the hybrid composite first increase. However, as the weight fraction of bamboo micro filler increases further (from 3 to 6 % in this work and from 5 to 12.5 % in previous work), the hybrid composite's tensile and flexural strengths begin to decrease.

**Table 3:** Mechanical and water absorption properties of hybrid composites.

Weight fraction of bamboo micro filler	Ultimate Tensile strength (MPa)	Ultimate Flexural strength (MPa)	References
0	83	106.3	Current work
3	87	112.6	
6	77	103.2	
0	26.5	33	(39)
5	34	52	
12.5	26	38	

## 4 Conclusions

Findings from tensile, flexural, impact and water absorption test allow us to draw the following conclusions:

✓ The tensile, flexural, impact, and water absorption characteristics of hybrid composites were significantly impacted by the weight percentage of bamboo fibers, sisal fibers, epoxy matrix, and bamboo micro filler.
✓ Based on tensile, flexural and impact tests, the result of maximum and minimum values of hybrid composites were observed at 3 % and 6%weight fraction of bamboo micro fillers respectively.
✓ With a 57 % epoxy matrix and 3 % bamboo micro filler, the hybrid composites contain 15 % Sisal, 10 % E-glass fibers, and 15 % bamboo fibers showed the highest tensile strength, maximum flexural strength, maximum impact strength, and toughness of 87 MPa, 112.6 MPa, 8.87 J, and 24.64 J/cm^2^, respectively.
✓ Additionally, a hybrid composite with 20 % sisal fiber, 10 % E-glass fiber, 10 % bamboo fiber, 10 % with 6 % bamboo fiber, and 54 epoxy matrix highlighted lower longitudinal tensile strength, lower flexural strength, and lower impact energy, which were found to be 77 MPa, 103.2 MPa, and 8.14J, respectively.
✓ Regarding water absorption capacity, maximum and minimum water absorption capacity were observed in hybrid composites of 20 % glass and 10 % glass fibers, 10 % bamboo fibers with a 54 % epoxy matrix and 6 % bamboo micro filler, and hybrid composites of 15 % glass fibers and 10 % glass fibers, 15 % bamboo fibers with a 57 % epoxy matrix and 3 % respectively

## CRediT authorship contribution statement

**Debela N. Gurmu:** Writing – review & editing, Visualization, Validation, Methodology, Investigation, Conceptualization. **Hailemariam M. Gebrelibanos:** Writing – original draft, Visualization, Methodology, Investigation, Conceptualization. **Chala A. Tefera:** Visualization, Validation, Investigation, Conceptualization. **Belete Sirahbizu:** Supervision, Methodology, Investigation, Conceptualization.

## Data availability statement

The data that support the findings of this study are available from the corresponding author upon reasonable request.

## Declaration of competing interest

The authors declare that they have no known competing financial interests or personal relationships that could have appeared to influence the work reported in this paper.
